# Inhibitory Activity of Natural *cis*-Khellactone on Soluble Epoxide Hydrolase and Proinflammatory Cytokine Production in Lipopolysaccharides-Stimulated RAW264.7 Cells

**DOI:** 10.3390/plants12203656

**Published:** 2023-10-23

**Authors:** Jang Hoon Kim, Ji Hyeon Park, Sung Cheol Koo, Yun-Chan Huh, Mok Hur, Woo Tae Park, Youn-Ho Moon, Tae Il Kim, Byoung Ok Cho

**Affiliations:** 1 Department of Herbal Crop Research, National Institute of Horticultural and Herbal Science, RDA, Eumseong 27709, Chungcheongbuk-do, Republic of Korea; oasis5325@gamil.com (J.H.K.); ksch992@korea.kr (S.C.K.); wmelon@korea.kr (Y.-C.H.); mok0822@korea.kr (M.H.); harusarinamu@korea.kr (W.T.P.); yhmoon@korea.kr (Y.-H.M.); coolkite@korea.kr (T.I.K.); 2 Institute of Health Science, Jeonju University, 303 Cheonjam-ro, Wansan-gu, Jeonju-si 55069, Jeollabuk-do, Republic of Korea; wlgusliza@naver.com

**Keywords:** *cis*-khellactone, soluble epoxide hydrolase, competitive inhibitor, molecular simulation, anti-inflammation

## Abstract

The pursuit of anti-inflammatory agents has led to intensive research on the inhibition of soluble epoxide hydrolase (sEH) and cytokine production using medicinal plants. In this study, we evaluated the efficacy of *cis*-khellactone, a compound isolated for the first time from the roots of *Peucedanum japonicum*. The compound was found to be a competitive inhibitor of sEH, exhibiting an IC_50_ value of 3.1 ± 2.5 µM and *k*_i_ value of 3.5 µM. Molecular docking and dynamics simulations illustrated the binding pose of (−)*cis*-khellactone within the active site of sEH. The results suggest that binding of the inhibitor to the enzyme is largely dependent on the Trp336–Gln384 loop within the active site. Further, *cis*-khellactone was found to inhibit pro-inflammatory cytokines, including NO, iNOS, IL-1β, and IL-4. These findings affirm that *cis*-khellactone could serve as a natural therapeutic candidate for the treatment of inflammation.

## 1. Introduction

*Cis*-5,8,11,14-Eicosatetraenoic acid [1], also known as arachidonic acid (AA), is an omega-6 polyunsaturated fatty acid, which is metabolized by cyclooxygenases, lipoxygenases, and cytochrome P450 enzymes to produce biologically fatty acid mediators [2]. In mammals, AA is obtained by directly ingesting AA-rich resources, or by ingesting linoleic acid, a precursor of AA, to biosynthesize AA [1]. Especially, AA is converted into epoxyeicosatrienoic acids (EETs) by cytochromeP450 enzyme [3]. EETs consist of four regioisomers: 5,6-EET, 8,9-EET, 11,12-EET, and 14,15-EET [4]. Known as endothelium-derived hyperpolarizing factors, EETs have demonstrated anti-inflammatory effects and benefits for cardiovascular health [5,6]. In particular, 11,12-EET and 14,15-EET have been observed to enhance vasodilator activity through large-conductance Ca^2+^-activated K^+^ channels in vascular smooth muscle cells [5]. 5,6- and 8,9-EETs have been reported to be potent angiogenic factors in vivo, promoting endothelial cell proliferation, migration, and development of capillary morphology [7]. In particular, among four EETs, 11,12- and 14,15 EETs are produced prominently [4]. Typically, ~40% of the EETs produced in the rat heart are 14,15-EET, and in the rat kidney, ~58% are 11,12-EET [4]. 11,12- and 14,15-EETs are found in human renal cortex and kidney [4]. Moreover, low concentrations of 8,9-EET, 11,12EET-, and 14,15-EET were found in lung cells of patients with idiopathic pulmonary fibrosis. It has been reported that transforming growth factor-induced expression of a-smooth muscle actin and collagen type-1 in MRC-5 cells, and primary fibroblasts decreases their concentration and progress by 11,12-EET, respectively [8]. EETs as anti-inflammatory activator downregulates NF-κB transcription factor induced by damage, stress, and inflammation [9]. EETs, known to suppress the expression of TNF-α and MCP-1 while inactivating IκB degradation, decrease in concentration due to the presence of sEH [10]. This leads to a loss of protection against cardiovascular and inflammatory diseases [10].

Soluble epoxide hydrolase (sEH, EC 3.3.2.10), encoded by the ephx2 gene, is a bifunctional homodimeric enzyme composed of 62.5 kDa monomers, each with a 35 kDa C-terminal hydrolase and a 25 kDa N-terminal phosphatase [11]. This enzyme is distributed throughout various tissues, including the aorta, heart, renal vasculature, liver, brain arterioles, lung, and adipose tissue [12,13]. Specifically, the C-terminal domain of sEH converts EETs into corresponding dihydroxyeicosatrienoic acids (DHETs), compounds with low bioactivity that are released from cells [4,10]. sEH, which has this function, has been considered a treatment target for inflammatory diseases [14]. Crystal structure studies have revealed the catalytic site of sEH and the catalytic triad involved in converting the epoxy ring to diol [9]. They are found to be Tyr382, Tyr465, and Asp335. Tyr382 and Tyr465 form hydrogen bonds with epoxide oxygen, and then epoxide carbon becomes active due to polarization, and then Asn334 participates nucleophilic interaction to epoxide [15]. As first-generation sEH inhibitors, compounds based on the skeleton of *trans*-3-phenylglycidols and chalcone oxides were developed [15]. Recently, urea-type derivatives were found to be potential sEH inhibitors [15]. To maintain the concentration of EETs, research into inhibiting the catalytic reaction of sEH is ongoing [16]. To overcome challenges such as the low solubility and poor metabolic stability of urea-type inhibitors [15], compounds like flavonoids from *Inula britanica* [17], triterpenoids from *Alisma orientale* [18], and amine-alkaloids from *Scutellaria baicalensis* [19] are under investigation.

Coumarin, a secondary metabolite found in many plants, was first isolated from *Coumarouna odorata* Aube [20], which has been called “tonka bean” [21]. Among its subtypes, angular pyranocoumarins comprise pyrano fused to coumarin [20,22]. (−)*cis*-Khellactone, an angular pyranocoumarin, has been reported from the rhizomes of *Angelica amurensis* [23] and Peucedani Radix [24]. This natural compound has demonstrated growth suppression of MCF7, MDAMB-231, and MCF10A cells depending on concentration and treatment duration [23]. Furthermore, *cis*-khellactone downregulated the expression of proinflammatory cytokines such as IL-23, TNF-a, IL-1β, and IL-6 in psoriatic skin [25]. (−)*cis*-Khellactone has been isolated from *Angelica amurensis* [23] and *Seseli devenyense Simonk* [26], and analyzed in Peucedani Radix [24]. Specifically, to determine the absolute configuration of (3′*S*,4′*S*)-3′-*O*-isobutyroyl-4′-*O*-(2*S*-methylbutyroyl)khellactone in *P. japonicum* roots, *cis*-khellactone was produced via alkaline hydrolysis of the compound [27]. Pharmacological efficacies of *cis*-khellactone have been shown to suppress the expression of IL-23, TNF-α, IL-1β, and IL-6 in psoriatic skin [25].

*Peucedanum japonicum* is a perennial plant belonging to Umbelliferae family and is mainly distributed in southern and eastern Asia [28]. Usually, this plant grows naturally on the coast [28]. This plant is cultivated by farmers as a vegetable [28,29]. The roots of *P. japonicum* has been known to be used for coughs and cold treatment as the traditional medicine [28,29]. Especially, the components of this plant have been revealed khellactone esters which have a biological activities, such as anti-oxidant, anti-inflammation, and anti-obesity [29,30,31]. The goal of this study was to evaluate the sEH inhibitory effect of *cis*-khellactone derived from the roots of *P. japonicum* and to explore its inhibitory effect on pro-inflammatory factors expressed in LPS-stimulated RAW264.7 cells.

## 2. Results

### 2.1. Compound Isolation and sEH Inhibitory Activity

**Figure 1:**
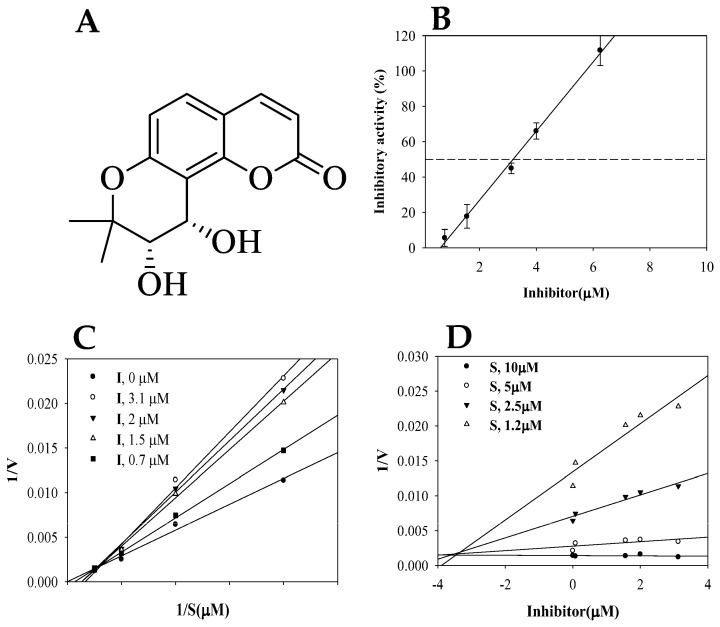
The structure of (−)*cis*-khellactone (**A**), and the inhibitory activity (**B**), Lineweaver–Burk (**C**), and Dixon (**D**) plots of inhibitor on sEH.

The roots of *P. japonicum* were subjected to ethanol extraction and subsequent partitioning using *n*-hexane and ethyl acetate. The ethyl acetate fraction was further processed through silica gel and C-18 column chromatography to yield (−)*cis*-khellactone for the first time. The chemical structure was identified by comparing Mass, and ^1^H-/^13^C-NMR spectra data with references in the literature (Figure 1A, Figures S1–S3) [23,27]. The isolated compound was evaluated for its ability to inhibit the catalytic conversion of non-fluorogenic substrate, (3-phenyl-oxiranyl)-acetic acid cyano-(6-methoxy-naphthalen-2-yl)-methyl ester (PHOME), to fluorescent 6-methoxy-2-naphthaldehyde and cyanide with sEH [32].

The inhibitory effect of the reaction between sEH and PHOME was evaluated at concentrations of (−)*cis*-khellactone ranging from 0.7, 1.5, 3.1, 4.0 and 6.2 µM. The inhibitory rates at each compound concentration were calculated using Equation (1). Additionally, the optimal Equation (2) across each inhibition rates was derived. These results are presented in Figure 1B. Commercial 12-(3-((3s,5s,7s)-adamantan-1-yl)ureido)dodecanoic acid (AUDA), used as a positive control, had an IC_50_ value of 0.0212 ± 0.3 µM. The inhibitory activity of the isolated compound was confirmed in a dose-dependent manner, presenting an IC_50_ value of 3.1 ± 2.5 µM (Figure 1B).

The inhibitor was prepared at concentrations ranging from 0.7 to 3.1 µM. The enzymatic reaction of sEH was then carried out with various substrate concentrations ranging from 1.2 to 10 µM at each inhibitor concentration. This procedure allowed for the calculation of initial values for the inhibitor, and subsequently, the derivation of Lineweaver–Burk and Dixon plots for sEH inhibition by the inhibitor. The resulting family of linear equations crossed a point of y-intercept (1/*V*_max_) and various x-intercepts (−1/*K*_m_) (Figure 1C). Therefore, it was concluded that the inhibitor is competitive in nature, with a *k*_i_ value calculated to be 3.5 µM by the Dixon plot (Figure 1D).

### 2.2. Molecular Docking between cis-Khellactone and sEH

**Figure 2:**
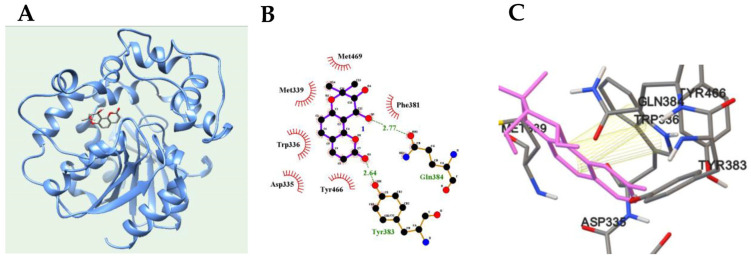
The best docking position (**A**), hydrogen bonds (**B**), and π–π (**C**) interactions of inhibitor.

Based on enzyme kinetic analysis, the inhibitor was confirmed to have high affinity for the catalytic site of sEH. The binding pose of the inhibitor enzyme was computed using the AutoDock 4.2 software package. The competitive inhibitor was docked into the grid containing the active site. As illustrated in Figure 2A and Table 1, the inhibitor was docked to the active site of sEH with an AutoDock score of −7.01 kcal/mol. Notably, the respective ketone and hydroxyl groups of the inhibitor maintained hydrogen bonds with Tyr383 (2.64 Å) and Gln384 (2.77 Å) (Figure 2B), while the benzene ring of the inhibitor maintained π–π interactions with the benzene (Å) and furan (Å) rings of Trp336 (Figure 2C).

### 2.3. Molecular Dynamics between cis-Khellactone and sEH

**Figure 3:**
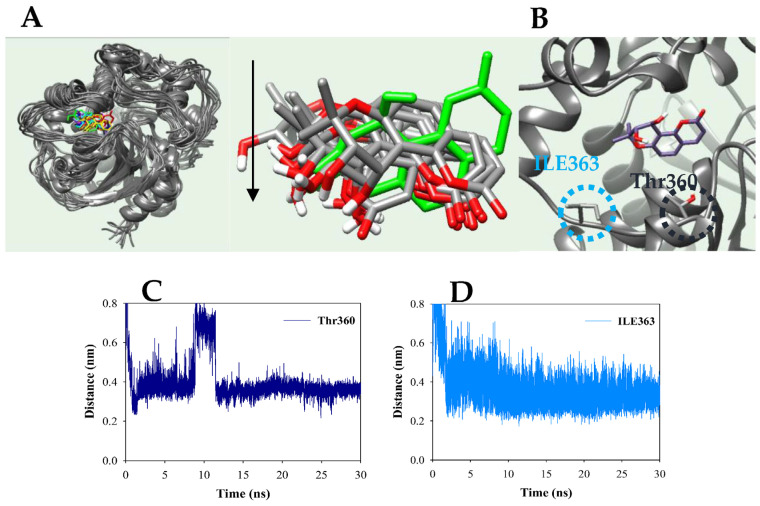
The superpositions of sEH with inhibitor for the simulation time (red: 0 ns; orange: 3 ns; yellow: 6 ns; green: 9 ns; cyan: 12 ns; blue: 15 ns; cornflower blue: 18 ns; purple: 21 ns; hot pink: 24 ns; magenta: 27 ns; black: 30 ns) (**A**). The distance of key residues (**B**) with inhibitor (**C**,**D**).

Molecular dynamics is a technique used to calculate the dynamic state of a ligand–receptor complex. This provides detailed information about ligand and receptor interactions beyond the limitations of molecular docking. Molecular dynamics was performed using the GROMACS 4.6.5 package, and intermolecular interactions were executed using the CHARMM force field. Molecular dynamics of the complex commenced at the end of the energy minimization, NVT, and NPT of the most stable docking. The results are depicted in Figure 3A,D and Figure S4A–D. The inhibitor was bound to a position (Pro371–Met469) slightly to the right of the active site. From an upright conformation at 0 ns, the inhibitor transitioned to a forward-tilted conformation of approximately 100 degrees from 3 ns to 30 ns (Figure 3A). The main core of the enzyme maintained the root mean square deviation (RMSD) values within about 3 Å with the potential energy of approximately −2.0 × 10^6^ kJ/mol during the simulation time (Figure S4A,B). The main core residues in sEH exhibited fluidity within 3 Å of the root mean square fluctuations (RMSF) values (Figure S4C). The ligand maintained 1–3 hydrogen bonds with the receptor (Figure S4D). Furthermore, key amino residues involved in hydrogen bonding with the (−)*cis*-khellactone-sEH were identified at 3 ns intervals during the simulation time (Figure 3B). The ketone of (−)*cis*-khellactone consistently maintained a 3.5 Å distance from Thr360, except between 0 and 0.8 ns and between 9 and 11.5 ns during the 30 ns simulation (Figure 3C). Similarly, the hydroxyl group of the inhibitor maintained a 3.5 Å distance with Ile362, except between 0 and 1.7 ns (Figure 3D).

### 2.4. Inhibition of NO Production and Pro-Inflammatory Cytokines by (−)cis-Khellactone in LPS-Stimulated RAW264.7 Cells

**Figure 4:**
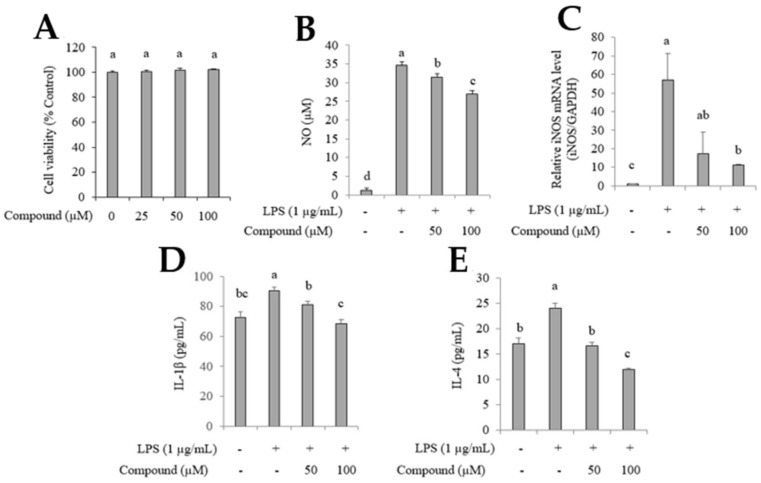
Effects of (−)*cis*-khellactone on cell viability and inhibition of NO production, iNOS mRNA levels, and the expression of IL-1b and IL-4. Cell viability (0, 25, 50, and 100 µM) was assessed via WST-8 assay (**A**). Cells were pretreated with (−)*cis*-khellactone for 1 h and then stimulated with LPS for 24 h, and the supernatant was obtained. NO production was performed by Griess reagent assay (**B**), and mRNA expression levels of iNOS was measured via real-time PCR (**C**). Expression of IL-1β (**D**) and IL-4 (**E**) were measured by ELISA assay. Each bar represents the mean ± SD. Different small case letters indicate significant differences at *p* < 0.05.

The anti-inflammatory activity of (−)*cis*-khellactone was evaluated in RAW264.7 cells and was found to not affect the viability of RAW264.7 cells at 25, 50, and 100 µM (Figure 4A). RAW264.7 cells were treated with (−)*cis*-khellactone (50 and 100 μM) for 2 h, then incubated with LPS for 24 h, and subsequently, the amount of NO production was measured. Results showed that 35.0 ± 0.4, 32.0 ± 0.2, and 27.4 ± 0.4 µM concentrations of NO were produced in cells treated with LPS, LPS with 50 µM inhibitor, and LPS with 100 µM inhibitor, respectively; 1.5 ± 0.4 µM NO was produced in normal cells (Figure 4B). Additionally, mRNA levels of iNOS (56.1-fold, 17.8-fold, and 10.8-fold) associated with NO production in LPS-treated cells were significantly suppressed by the inhibitor (50 and 100 µM) in a dose-dependent manner compared to LPS-untreated cells (Figure 4C). The inhibitory activity of (−)*cis*-khellactone on the expression of cytokines in LPS-stimulated RAW264.7 cells was also evaluated. As depicted in Figure 4D,E, the levels of IL-1β and IL-4 in LPS-stimulated RAW264.7 cells treated with the inhibitor at 50 µM (IL-1β: 82.0 ± 3.0 pg/mL; IL-4: 16.7 ± 0.2 pg/mL) and 100 µM (IL-1β: 67.8 ± 3.4 pg/mL; IL-4: 11.9 ± 0.1 pg/mL) were reduced compared to cytokine levels in LPS-stimulated RAW264.7 cells (IL-1β: 89.0 ± 0.7 pg/mL; IL-4: 24.1 ± 0.4 pg/mL). The levels of IL-1β and IL-4 in RAW264.7 cells (LPS-untreated cells) were 74.5 ± 0.5 pg/mL and 17.1 ± 0.5 pg/mL, respectively.

## 3. Discussion

In the present study, we isolated (−)*cis*-khellactone from *P. japonicum* roots for the first time, confirming its structure by analyzing Mass and NMR signals. The compound (purity over 96%) displayed concentration-dependent inhibition of PHOME hydrolysis by sEH. Consequently, the IC_50_ value of the inhibitor was determined to be 3.1 ± 2.5 µM. Furthermore, this compound was found to bind the active site of sEH with *k*_i_ value of 3.5 µM. Recent studies on sEH inhibitors have demonstrated that flavonoids [33] and sesquiterpenes [34] from natural plants exhibit significant efficacy. Specifically, coumarin derivatives, such as omphalocarpin, murrangatin, and kimcuongin from *Murraya paniculata* in Vietnam, have shown sEH inhibitory effects [35].

A molecular simulation tracked the binding between (−)*cis*-khellactone and the catalytic site in sEH, while molecular docking confirmed that hydroxyl and ketone groups of (−)*cis*-khellactone maintained hydrogen bonds between Gln384 and Tyr383, respectively, and π–π interactions with Trp336. In addition, molecular dynamics showed that the hydroxyl and ketone groups of (−)*cis*-khellactone maintained a steady 3.5 Å distance from lle363 and Thr360, respectively. This suggests that *cis*-khellactone binding to sEH relies on movement of the Trp336–Gln384 loop. However, EETs are converted to DHETs via interaction with Asp333, Asp495, and His523 of the epoxide hydrolase catalytic triad residues [36]. As mentioned above, it was confirmed that (−)*cis*-khellactone maintains the binding to sEH by using different amino residues instead of catalytic triad that interact with the substrate. Therefore, the development of sEH inhibitors using a khellactone moiety should consider the amino residue of the loop proposed above. Additionally, this inhibitor demonstrated anti-inflammatory activity by downregulating NO production, iNOS mRNA levels, and IL-1β and IL-4 expression at 50 and 100 µM. Hence, (−)*cis*-khellactone was confirmed to function as an anti-inflammatory agent through sEH and proinflammatory inhibition.

## 4. Materials and Methods

### 4.1. General Experimental Procedures

The confirmation of compounds separation was conducted using thin-layer chromatography (TLC) on pre-coated silica gel 60 F254 and silica gel RP-18 F254 glass plates (20 × 20 cm; Merck, Darmstadt, Germany), and confirmed under 254 nm ultraviolet light. An ethanol solution containing 10% sulfuric acid was used to color the compounds in TLC. Open column chromatography was performed with 230–400 mesh silica gel 60 (Merck, Darmstadt, Germany) and 12 nm and S–75µm ODS-A (YMC, Kyoto, Japan). The purity of the compound was confirmed through signals performed in the agilent UHPLC 1290 infinity Ⅱ system (Santa Clara, CA, USA). Nuclear magnetic resonance (NMR) signals were generated during analysis on Bruker Avance III 400 MHz spectrometers (Bruker, Billerica, MA, USA). The molecular weight signal was obtained with an LCMS-2020-EV equipped with electrospray ionization (Shimadzu, Kyoto, Japan). The reagents for buffer (tris, B9754; bovine serum albumin (BSA), A8806) were purchased from the professional reagent store (Sigma-Aldrich, St. Louis, MO, USA). Mammalian recombinant sEH (10011669), PHOME (10009134), and AUDA (10007972) were sourced from Cayman Chemical (Ann Arbor, MI, USA). The Cell Viability Assay Kit was obtained from BIOMAX (Guri, Republic of Korea). Lipopolysaccharides (LPS) and Griess reagent were procured from Sigma-Aldrich. IL-1β and IL-4 enzyme-linked immunosorbent assay (ELISA) kits were purchased from R&D Systems (Minneapolis, MN, USA).

### 4.2. Plant MATERIAL

The roots of *P. japonicum* Thunberg were produced and harvested at a farmhouse on Geumod Island, Jeollanam-do, Republic of Korea, on 30 March 2021. The species was identified by Dr. J.H. Kim. A voucher specimen (PJR210330) was deposited with the herbarium of the Department of Herbal Crop Research, National Institute of Horticultural and Herbal Science. The collection of this plant was complied with relevant institutional, national, and international guidelines and legislation.

### 4.3. Extract and Isolation

The roots of *P. japonicum* (3 kg) were extracted three times with ethanol (18 L) at room temperature. The concentrated extracts (120 g), obtained under reduced pressure, were suspended in distilled water (7 L) and then successively partitioned with *n*-hexane and ethyl acetate, yielding *n*-hexane (85 g) and ethyl acetate (13 g) fractions. The ethyl acetate fraction was subjected to silica gel open column chromatography (Φ 7 × 20 cm) with a gradient solvent system of CHCl_3_-MeOH (20:1 → 7:1), resulting in seven fractions (E1–E7). Fraction E5 (72 mg) was chromatographed on open C-18 column (Φ 3 × 40 cm) with an isocratic solvent system of H_2_O-MeOH (2:3) to yield the compound of interest (10 mg).

### 4.4. Spectrua Assignment of cis-Khellactone

Compound. Oily, ${\left[\alpha \right]}_{D}^{22}$ −16.0° (CDCl_3_, *c* 0.001), ESI-MS: *m*/*z*: [M + H]^+^ *m*/*z* 263.05, ^1^H (400 MHz, CDCl_3_) *δ* 7.66 (d, *J* = 9.5 Hz, 1H), 7.32 (d, *J* = 8.6 Hz, 1H), 6.79 (d, *J* = 8.6 Hz, 1H), 6.25 (d, *J* = 9.5 Hz, 1H), 5.19 (d, *J* = 4.9 Hz, 1H), 3.85 (s, 1H), 1.45 (s, 3H), 1.41 (s, 3H).^13^C-NMR (100 MHz, CDCl_3_): *δ*_C_ 161.3 (C-2), 156.5(C-7). 154.6 (C-9), 144.5 (C-4), 128.7 (C-5), 115.0 (C-6), 112.3 (C-10), 112.1 (C-8), 111.0 (C-3), 79.1 (C-2′), 71.1 (C-3′), 61.1 (C-4′), 25.1 (C-CH_3_), (21.7 C-CH_3_).

### 4.5. sEH Enzymatic Assay

This study was carried out as previously described [33]. To determine inhibitory activity, 130 µL of diluted sEH in buffer (25 mM bis-Tris-HCl, pH 7.0) containing 0.1% BSA was mixed to 20 µL of either the (−)*cis*-khellactone dissolved in MeOH or MeOH alone. Then, 50 µL of the substrate in buffer (PHOME) was mixed to each reagent solution and heated at 37 °C to initiate sEH hydrolysis. The resultant output was monitored at an excitation of 330 nm and an emission of 465 nm within an hour. The percentage inhibition was calculated as follows:

Percentage inhibition (%) = [(control result − experimental result)/control result] × 100(1)

where the following are defined:

Control result: The measured result in the absence of the inhibitor.

Experimental result: The measured result in the presence of the inhibitor.

y = y_0_ + [(a × x)/(b + x)] (2)

y_0_: Initial values of “y” when “x” is zero. “a” denotes the difference between maximum and minimum values, and “b” refers to the “x” value at 50%.

### 4.6. Molecular Docking

Molecular docking was conducted as previously described [33]. (−)*cis*-Khellactone with a 3D structure was constructed and minimized using Chem3D Pro (Cambridge Soft, Cambridge, MA, USA). The protein 3D structure of the receptor, coded as 3ANS, was downloaded from the RCSB Protein Data Bank (https://www.rcsb.org, accessed on 15 August 2023). As only the A-chain of this receptor (sEH) was selected for docking, the other was excluded. Water and ligand (4-cyano-*N*-[(1*S*,2*R*)-2-phenylcyclopropyl]-benz- amide) were also deleted in the A-chain.

A-chain added with hydrogen by AutoDockTools (Scripps Research, La Jolla, CA, USA) was applied with Gasteiger charge. Flexible (−)*cis*-khellactone simulation was performed using a torsion tree, detecting the torsion root and rotatable bonds. The grid box was set to a size of 60 × 60 × 60 (center grid box: x, 24.612; y, 26.057; z, 117.11) for docking (−)*cis*-khellactone into the active site. Molecular docking was achieved using a Lamarckian genetic algorithm with the maximum number of evaluations. The resulting values were calculated and visualized using AutoDockTools, Chimera 1.14 (San Francisco, CA, USA), and LIGPLOT (European Bioinformatics Institute, Hinxton, UK).

### 4.7. Molecular Dynamics

The molecular dynamics study was performed with slight modifications from the method described previously [33]. Molecular dynamics analysis was conducted using the Gromacs 4.6.5 package to simulate the sEH complex with (−)*cis*-khellactone, which is charged using a CHARMM all-atom force field. The inhibitor’s str file, generated by the GGenFF server, was converted to gro. and itp. files with CHARMM36-ff. The charged sEH-(−)*cis*-khellactone complex was dissolved in water and six sodium ions within a cubic box using the simple point charge water model. Mdp files were generated in accordance with GROMACS guidelines. The mdp files were minimized to a maximal force of 10 kJ/mol using the steepest-descent method. The result data was sequentially equilibrated at 300 K NVT in 1 bar NPT for 100 ps. Finally, a molecular dynamics simulation was conducted for 30 ns. The results were analyzed using gutility. Data were visualized using SigmaPlot 10.0 (Systat Software Inc., San Jose, CA, USA) and Chimera.

### 4.8. Cell Culture and Viability

RAW264.7 macrophages were cultured in a CO_2_ incubator for 24 h after seeding into a 96-well plate at a concentration of 2 × 10^5^ cells/mL in DMEM media, supplemented with 10% FBS and 1% penicillin/streptomycin. The cells were then treated with (−)*cis*-khellactone (at concentrations of 25, 50, and 100 µM) for 24 h. Subsequently, 10 μL of water-soluble tetrazolium salt (WST-8) was added to each well, and after incubation for 4 h, absorbance was measured at 450 nm with a spectrophotometer (Tecan Group, Ltd., Mannedorf, Switzerland).

### 4.9. Nitric Oxide Assay

RAW264.7 macrophages were cultured at a density of 2 × 10^5^ cells/mL in 96-well plates for 24 h. The cells were then pre-treated with (−)*cis*-khellactone (at concentrations of 50 and 100 µM), and after 2 h, stimulated with 1 µg/mL of LPS for 24 h. Following incubation, 100 µL each of supernatant and Griess reagent were added to the 96-well plates. Absorbance was then measured at 450 nm using a spectrophotometer (Tecan Group, Ltd., Mannedorf, Switzerland).

### 4.10. Enzyme-Linked Immunosorbent Assay

RAW264.7 macrophages were cultured in 6-well plates at a density of 2 × 10^5^ cells/mL for 24 h and then pre-treated with (−)*cis*-khellactone (at concentrations of 50 and 100 µM). After 2 h, cells were stimulated with 1 µg/mL of LPS for 24 h. Post-stimulation, cell culture supernatants were collected, and IL-4 and IL-1β cytokine levels were measured using the ELISA kit from R&D Systems according to the manufacturer’s protocol.

### 4.11. RNA Extraction and Real Time-PCR

RAW264.7 macrophages were cultured in 6-well plates at a concentration of 2 × 10^5^ cells/mL and incubated for 24 h. They were then pre-treated with (−)*cis*-khellactone (50 and 100 µM) for 2 h, followed by stimulation with LPS for 3 h. Total RNA was extracted using an RNA-spin™ Total RNA Extraction Kit (iNtRON Biotechnology, Seongnam, Republic of Korea) and reverse-transcribed into cDNA using an iScript™ cDNA Synthesis Kit and a T100TM Bio-Rad Thermal Cycler (Bio-Rad, Hercules, CA, USA). The resultant cDNAs were amplified using a SYBR kit (TOYOBO Co., Ltd., Osaka, Japan). Real-time PCR was performed on a StepOne Real-Time PCR system (Thermo, Waltham, MA, USA) and gene expression levels were quantified. Primers used for PCR analysis were as follows: iNOS, 5′-CGGAGTGACGGCAAACATGA-3′ (forward) and 5′-TTCCAGCCTAGGTCGATGCA-3′ (reverse); GAPDH, 5′-GGCTACACTGAGGACCAGGT-3′ (forward) and 5′-TCCACCACCCTGTTGCTGTA-3′ (reverse). The thermal profile included 95 °C for 5 min, followed by 30 cycles of amplification at 95 °C for 30 s and 60 °C for 30 s. Expression levels were normalized to GAPDH using the 2^−ΔCt^ method. All procedures adhered to the manufacturer’s instructions without any modifications.

### 4.12. Statistical Analysis

All measurements were conducted in triplicate across three independent experiments, with results displayed as the mean ± standard error of the mean (SEM). Data analysis was performed using SigmaPlot 10.0 software. The statistical significance was set at *p* ≤ 0.05.

## 5. Conclusions

(−)*cis*-Khellactone, isolated from *P. japonicum* roots through open column chromatography, was found to inhibit sEH with an IC_50_ value of 3.1 ± 2.5 µM. The competitive inhibitor with a *k*_i_ value of 3.5 µM was calculated through molecular simulations to stably bind the Trp336–Gln384 loop in the active site of sEH. Furthermore, *cis*-khellactone inhibited NO production, iNOS mRNA levels, and the expression of IL-1β and IL-4 in LPS-stimulated RAW267.7 cells. This study thus demonstrates the potential of (−)*cis*-khellactone as a candidate for the development of anti-inflammatory drugs by inhibiting sEH and proinflammatory cytokines.

## Figures and Tables

**Table 1:** The inhibitory activity, kinetics, and molecular docking of inhibitor with sEH.

	IC_50_ (μM) ^a^	Binding Mode (*k*_i_, μM)	Autodock Score (kcal/mol), Hydrogen Bonds
1	3.1 ± 2.5	Competitive (3.5)	−7.01, Tyr383 (2.64), Gln384 (2.77)
AUDA ^b^	0.021.2 ± 0.3		

^a^ Compound examined a set of triplicated experiment. ^b^ Positive control.

## Data Availability

All data are included in the main text and Supplementary Materials.

## Supplementary Materials

The following supporting information can be downloaded at: https://www.mdpi.com/article/10.3390/plants12203656/s1, Figure S1: UV-Vis (A), HPLC (B) and Mass (C) spectrua of *cis*-khellactone.; Figure S2: ^1^H NMR spectrum of *cis*-khellactone (400 MHz, CDCl_3_-*d*).; Figure S3: ^13^C NMR spectrum of *cis*-khellactone (100 MHz, CDCl_3_-*d*).; Figure S4: The potential energy (A) RMSD (B), RMSF (C), and hydrogen bond numbers (D) of the simulation calculated during 30 ns.
