# Supplementary material for: Inhibitory Activity of Natural cis-Khellactone on Soluble Epoxide Hydrolase and Proinflammatory Cytokine Production in Lipopolysaccharides-Stimulated RAW264.7 Cells
Source: Plants (Basel). 2023 Oct 23;12(20):3656. doi: 10.3390/plants12203656 (PMC10610198; doi:10.3390/plants12203656)
Supplement: Supplementary file 1 [file plants-12-03656-s001.zip › plants-2634165-supplementary.pdf]

plants

MDPI

Supplementary Material

Inhibitory activity of natural *cis*-khellactone on soluble epoxide hydrolase and proinflammatory cytokine production in LPS-stimulated RAW264.7 cells

Jang Hoon Kim[1], Ji Hyeon Park[2], Sung Cheol Koo[1], Yun-Chan Hur[1], Mok Hur[1], Woo Tae Park[1], Youn-Ho Moon[1], Tae Il Kim[1], Byoung Ok Cho[2,*]

[1]Department of Herbal Crop Research, National Institute of Horticultural and Herbal Science, RDA, Eumseong, Chungcheongbuk-do, 27709, Republic Korea

[2]Institute of Health Science, Jeonju University, Jeonju-si, Jeollabuk-do, 55069, Republic of Korea

*** Correspondence:**
Corresponding Author : Byoung Ok Cho, Institute of Health Science, Jeonju University, 303 Cheonjam-ro, Wansan-gu, Jeonju-si, Jeollabuk-do, 55069, Republic of Korea

Tel: +82-63-220-2793, Fax: +82-63-220-2789, E-mail: enzyme21@jj.ac.kr

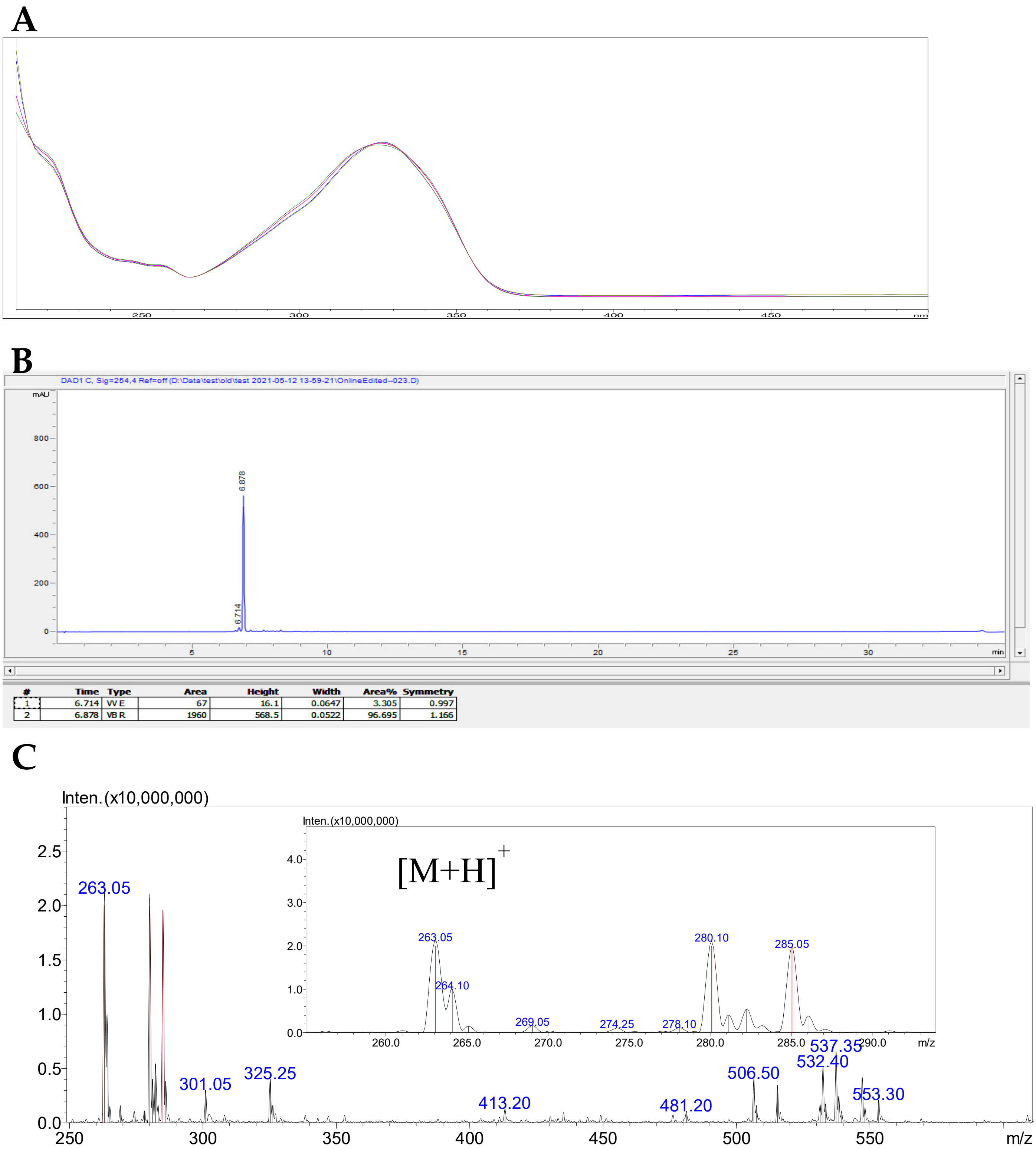

Figure S1. UV-Vis (A), HPLC (B) and Mass (C) spectrua of *cis*-khellactone.

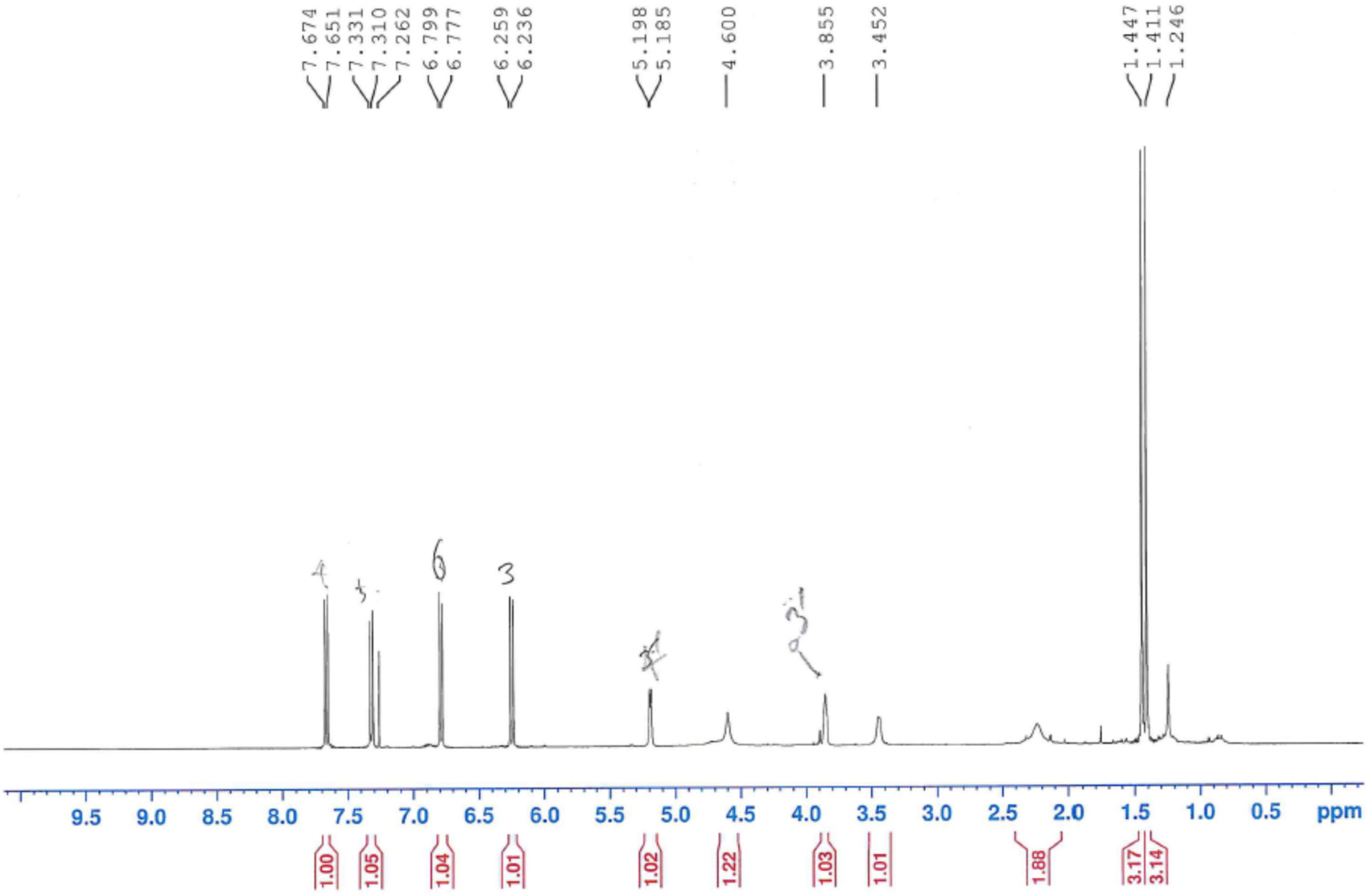

Figure S2. ^{1}H NMR spectrum of *cis*-khellactone (400 MHz, $CDCl_3$-*d*).

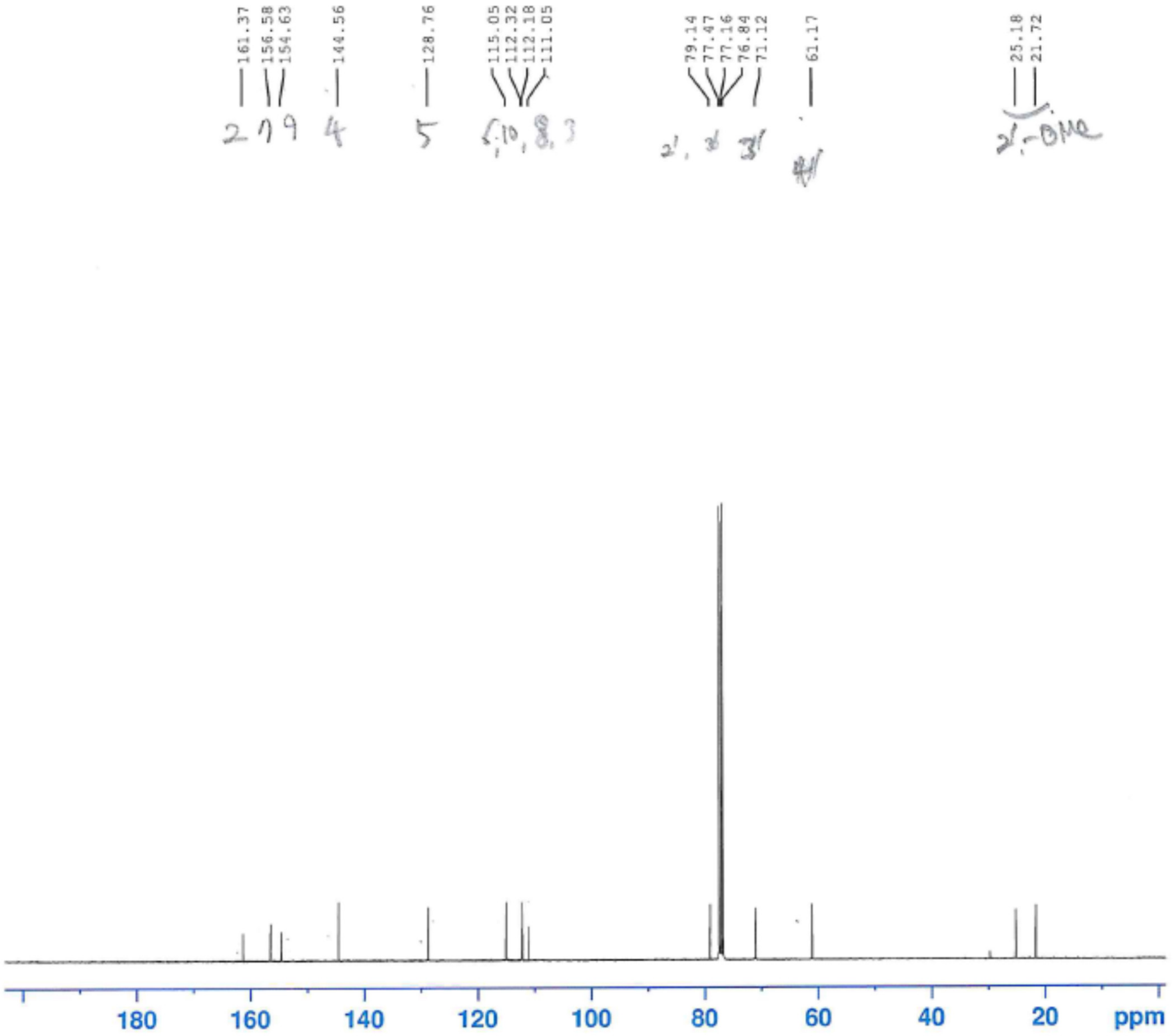

Figure S3. ^{13}C NMR spectrum of *cis*-khellactone (100 MHz, $CDCl_3$-*d*).

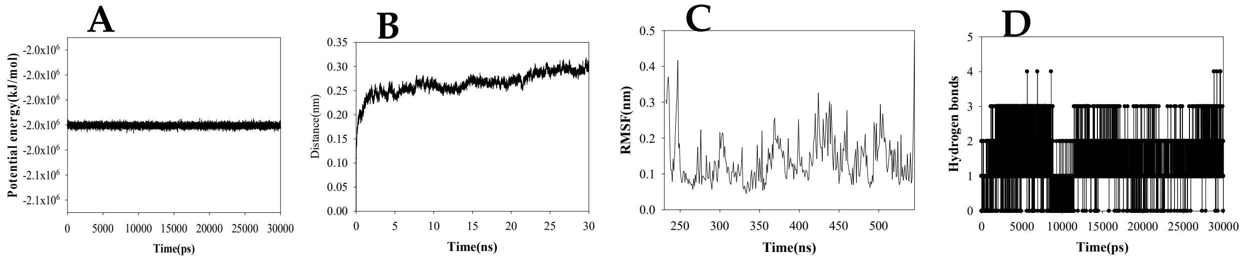

Figure S4. The potential energy (A) RMSD (B), RMSF (C), and hydrogen bond numbers (D) of the simulation calculated during 30 ns.
